# The value, challenges and practical considerations of conducting qualitative research on antimicrobial stewardship in primary care

**DOI:** 10.1093/jacamr/dlac026

**Published:** 2022-03-16

**Authors:** Marta Wanat, Marta Santillo, Aleksandra J. Borek, Christopher C. Butler, Sibyl Anthierens, Sarah Tonkin-Crine

**Affiliations:** 1 Nuffield Department of Primary Care Health Sciences, University of Oxford, Oxford, UK; 2 Department of Family Medicine and Population Health, University of Antwerp, Antwerp, Belgium; 3 National Institute for Health Research Health Protection Research Unit in Healthcare Associated Infections and Antimicrobial Resistance, Oxford, UK

## Abstract

In order to design appropriate antimicrobial stewardship (AMS) programmes, it is crucial to understand challenges to tackling antibiotic resistance (AMR) specific to each healthcare setting. Antibiotic prescribing in primary care accounts for most prescriptions with a significant proportion considered clinically inappropriate. Qualitative research has a long history in social sciences, but its value and contribution are still contested in medical journals including in the AMR/AMS field. However, through its focus on understanding, meaning making and explaining, qualitative research can offer insights in how to improve AMS efforts in primary care. This paper provides an overview of unique considerations, contributions and challenges related to using qualitative research in AMS to help the AMS community new to qualitative research to utilize its potential most fully. First, we discuss specific considerations for AMS in relation to the stages of conducting a qualitative study, including identifying a research question and choosing a suitable methodology; sampling appropriate participants; planning a recruitment strategy; choosing a method of data collection; and conducting data analysis. These are illustrated with examples of qualitative AMS studies in primary care. Second, we highlight the importance of patient and public involvement throughout all stages of the project and ensuring quality in qualitative AMS research. Finally, drawing on these considerations, we make a further case for the value and contribution of qualitative methodologies in AMS/AMR research while outlining future directions for both AMS and qualitative research, including the need for studies with diverse actors; interdisciplinary collaborations; and complex decisions on methodologies and timelines.

## Introduction

### AMS and primary care

Antimicrobial resistance (AMR) occurs naturally but has been accelerated by human factors, including poor infection control, global travel and misuse of medicines.^1^ The latter involves not using antimicrobials as directed, using them when there is little or no benefit, or taking the wrong kind and in inappropriate concentrations.^1^ AMR has serious consequences; recent estimations show that 10 million lives a year are at threat due to the risk of drug-resistant infections unless solutions are found to slow this process.^2^

While there is a need for a One Health approach by understanding antibiotic use and preserving usefulness of antibiotics in all sectors,^3^ it is also important to understand challenges specific to each setting,^4^ including primary care. Antibiotic prescribing in primary care accounts for most antibiotic prescribing. For example, in England over 70% of antibiotics have been prescribed in primary care,^5^ with at least 20% of all antibiotic prescriptions considered clinically inappropriate.^1^ While great progress has been made in developing and using antimicrobial stewardship (AMS) programmes in primary care,^5^ in order to move the AMS field forward, we still need to address numerous challenges and priorities, including reducing the burden of infections, optimizing the use of antimicrobials and developing and using (new) diagnostics, therapies, vaccines and interventions.^1,2^ Qualitative research can make an important contribution in addressing these.

### Qualitative research and AMS

While having a long history in social sciences,^6^ qualitative research has slowly carved a space within primary care research. Great progress has been made in welcoming qualitative studies in medical journals^7^ with some highlighting that qualitative research methods are now an ‘intrinsic aspect of primary care research’.^8^ However, as described by van den Bergh and Brink^9^ and by others,^10,11^ its place and value can still, at times, be contested or simplified. For example, tensions feature in many discussions around qualitative research, such as whether qualitative studies should offer generalizable findings,^10^ whether samples should and could be representative,^10^ or about the value of information on philosophical underpinnings of qualitative studies.^10^ Thus, it is worth looking closely at what we mean by qualitative research. Aspers and Corte^12^ highlighted the distinctive features of being ‘qualitative’ as: understanding, interpretation, getting close and making distinction, while Willig^13^ described the key features as the focus on meaning, the attempt to describe and explain events and the need for interpretation.

Given its focus on understanding, meaning making and explaining, qualitative research can make a variety of contributions to AMS. In this paper, we provide an overview of unique considerations and challenges related to using qualitative research in primary care AMS to help the AMS community, including clinicians, researchers, policymakers and commissioners, to fully utilize its potential in research and in translating knowledge into policy and practice. We discuss these considerations in relation to each stage of a qualitative study. We draw on published examples of qualitative research in primary care on AMS (which are not exhaustive), with the aim of sharing good practice while also highlighting areas or methods that are underutilized or need further attention. We hope that this will provide a useful ‘how-to’ guide in applying qualitative methods for researchers new to qualitative research in the AMS field, which in turn will encourage them to adopt this approach or allow the AMS community to be more confident in reading and interpreting qualitative papers. We conclude with a discussion on quality in qualitative research, future directions for AMS qualitative research and our recommendations for taking qualitative AMS research forward.

## Designing and conducting qualitative research in AMS: important considerations

### Identifying relevant research questions and choosing the right methodology

While an epidemiological study may assess, for example, the rate of prescribing of antibiotics for respiratory tract infections (RTI) in out-of-hours settings, a qualitative study may focus on explaining these rates by exploring patients’ expectations when consulting out-of-hours or any difficulties clinicians face in managing patients with RTIs in out-of-hours settings. Thus, in quantitative studies we are likely to see questions focused on ‘how many, how much and how often?’ and in qualitative focused on ‘what, how and why?’^13,14^ Table 1 provides various examples of research questions in published qualitative studies on AMS and AMR in primary care.

**Table 1.** Examples of research questions which qualitative studies may address

	Questions
Focused on patients	What are parents’ perceptions and understanding of antibiotic use and resistance in the context of their young child with an acute RTI?^15^ What are the factors that shape migrants’ experiences of and attitudes to antibiotics in primary care?^16^ What are patients’ experiences of consulting a GP in a trial for treating acute cough?^17^
Focused on healthcare professionals	What are the views of professionals from high-prescribing practices about uptake and implementation of delayed prescribing and point of care C-reactive protein testing to reduce antibiotic use?^18^ What are the specific challenges when prescribing or dispensing antibiotics by general practitioners and pharmacists in out-of-hours primary care?^19^ What are clinicians’ views of the non-clinical factors that shape antibiotic prescribing decisions for lower RTI?^20^
Focused on policymakers and commissioners	What are the experiences of professionals from Clinical Commissioning Groups and general practices in England of implementing the Quality Premium quality incentive scheme, and their views on the Quality Premium’s role in influencing antibiotic prescribing?^21^ What are the conditions for the municipality chief medical officer’s involvement in quality improvement in general practice in relation to antibiotic prescribing?^22^

There is a variety of methodologies and methods that qualitative research encompasses. In fact, some researchers have highlighted the importance of referring to different qualitative ‘methodologies’ rather than ‘methodology’^13^ and the need to remember that qualitative research is a diverse field.^23^ Taking a broad-brush approach, qualitative approaches can be divided into experiential, thus focused on understanding people’s views and experiences, and discursive, thus interested in understanding how language can be used to construct a particular version of reality.^24^ Experiential methodologies include grounded theory, phenomenological approaches, narrative analysis or thematic analysis (among others), while discourse analysis and conversation analysis fall into the latter category. These different analytic methodologies come with unique sets of epistemological underpinnings, sampling approaches, associated methods of data collection and analysis. While we do not attempt to describe these differences here, and refer the readers to numerous textbooks (e.g.^13,25,26^), it is important to reflect how these approaches may offer different insights on AMS. Experiential approaches can be used in variety of ways, with a focus on understanding people’s perceptions of an issue; for example, they can help to understand patients’ experiences of having symptoms of an infection, how they manage it and what triggers their help seeking;^27^ or they can provide insights into patients’ experiences of a consultation.^28,29^ When used in studies with healthcare professionals (HCPs), they can highlight, for example, what HCPs found difficult when managing infections^30^ or their views of AMR and its influence on their antibiotic prescribing.^31^ Discursive approaches can equally be used in various ways, for example, describing and identifying interactional communication practices of patients and clinicians during consultations in primary care;^32–34^ understanding how patients may convey ‘pressure’ on clinicians to prescribe antibiotics and how clinicians react to these pressures;^35^ or identifying strategies that can help clinicians to respond to perceived requests for antibiotics.^35^

While the experiential and discursive approaches bring different perspectives and insights, these insights are often complementary. In fact, in some studies they have been combined; for example, Cabral *et al.* used conversation analysis to examine communication practices between parents and clinicians (i.e. how they communicated with each other rather than what they thought of this communication)^33^ and combined this with an experiential approach using interviews with parents,^36^ supported by watching the video of their consultation.^37^ These studies are relatively uncommon, as they require research teams to have expertise in distinctive approaches, but can offer powerful complementary perspectives. Understanding the differences between different methodologies is key not only to ensuring that an appropriate approach is chosen but also to expanding the repertoire used by primary care researchers, in general and in relation to AMS, to study it from different angles.

### Sampling and recruiting the right participants

AMS refers to efforts and approaches to support responsible use of antibiotics, thus it is important to define target samples carefully and purposefully. Table 2 highlights diversity of sampling and recruitment strategies possible for AMS qualitative studies. First, target populations are likely to include patients with experience of specific infections, including lower and upper RTIs, urinary tract infections (UTIs) or skin infections.^28,37,38^ Sampling here is likely to be based on their recent experience of a consultation but may also be based on whether patients had (or not) an antibiotic prescribed for that particular episode.^15^ Patients with recent experience of infections can be identified and recruited through primary care organizations. The heterogeneity of practices can be of importance here, based on specific study selection criteria, such as practice population size, patient and healthcare professional ethnic diversity, area and individual levels of deprivation, practice geographical location or antibiotic prescribing rates. For example, researchers may focus on understanding challenges facing high prescribing practices and recruit from only practices meeting this criterion;^18^ or they may want to understand contextual factors across low, medium and high prescribing practices and thus recruit from a diverse range of practices in relation to these criteria.^30^ It is important to consider these factors in the recruitment phase as they could affect the prescription of antibiotics and patient care in general.

**Table 2.** Examples of sampling and recruitment strategies in the published AMS studies in primary care

Study	Research question	Sampling	Recruitment strategy
Van Hecke *et al*.^15^	What are parents’ perceptions and understanding of antibiotic use and resistance in the context of their young child with an acute RTI?	Parents/carers aged 18 years; sampling three different groups: (i) parents who did not attend a healthcare facility; (ii) parents who consulted in primary or ambulatory care and were not prescribed an antibiotic; (iii) those who consulted and were prescribed an antibiotic	(i) recruitment via general practices: parents to be identified during routine consultation with either a GP or a nurse practitioner; (ii) recruitment via parent baby/toddler groups (researcher attending the group); (iii) recruitment via social media
Van der Zande *et al*.^30^	What are the contextual factors related to GPs’ antibiotic prescribing behaviour in low, high, and around the mean (medium) prescribing primary care practices?	Practices selected from North-West England in either bottom 10% (low prescribing); top 10% (high prescribing) and around the mean of the prescribing rate (medium prescribing); sampling within practices focused on GPs (no additional criteria)	(i) recruitment of practices directly by the researchers; (ii) recruitment of practices using Clinical Research Networks (organizations in England facilitating recruitment); (iii) GPs from practices also asked to make suggestions about potential participants
Lecky *et al.*^38^	What are the barriers to effective communication and antibiotic prescribing from the perspective of patients and GPs?	Women aged >16 years with experience of UTI in the last 12 months who consulted a GP regarding their symptoms; GPs with previous experience of consultations with women who had UTI	(i) patients recruited via the PHE People’s Panel (comprising members of the public); (ii) GPs invited through a clinical newsletter for the Royal College of General Practitioners
Høye *et al*.^22^	What are the conditions for the MCMOs’ involvement in quality improvement in general practice in relation to antibiotic prescribing?	MCMOs with responsibility for communicable disease control	(i) recruitment via independent organizations for MCMOs and the Norwegian Community medicine Association; (ii) attendees at biannual conference for MCMOs

MCMOs, municipality chief medical officers.

Second, studies can also focus more generally on patient and public understanding of AMR and/or AMS, and on unpacking beliefs about benefits and harms of antibiotic use and the perceived link between patients’ antibiotic use and resistance.^15,39,40^ Sampling here may involve patients with current infections (e.g. as part of discussions about consultation experience^41^), but also patients who had a (recent) experience,^15,40^ or no experience at all of a specific infection.^42^ In addition to recruitment through general practices, members of the public can be recruited through community organisations,^15,29^ and social media, which could provide access to additional specific subgroups of people. Specific recruitment strategies may need to be considered to ensure a successful recruitment of underrepresented groups, such as those from ethnic groups or economically deprived areas. These strategies may involve gaining support from leaders of community churches or relevant community organizations who can act as gatekeepers.^15^

Third, an important group of participants includes primary care HCPs. This is crucial to seek their views in order to identify their needs and support them better. This is also a heterogeneous group and participants can be sampled according to their professional role (e.g. doctors, nurses, pharmacists) but also their prescribing capability or responsibilities (e.g. for local AMS initiatives, quality improvement). For example, in the UK around 30 000 nurses and 4000 pharmacists^43^ have the same prescribing capability as GPs and are responsible for around 8% of all primary care antibiotics; these roles are gradually being integrated into primary care teams across the UK,^44^ and qualitative studies are also starting to explore their views, roles and behaviours. Fourth, AMS efforts involve policymakers and commissioners at both local and national level^21,45,46^ as antibiotic prescribing is not only influenced by individual factors but also norms and societal beliefs, wider contextual and policy influences.^46^ Sampling these stakeholders, for example, has helped understand how local policymakers would benefit from clearer roles and responsibilities in order to fully contribute to AMS.^22^ HCPs and policymakers can also be recruited from primary care through directly approaching their places of work.^31,46^ Other common strategies may include using professional organizations, networks and groups,^45,47^ using general advertising in publications or websites read/accessed by the target HCPs^48^ or using informal professional networks (e.g. social media groups and informal professional mailing lists).

For all these groups, study advisory groups (committees set up at the start of a project), which often include patients and clinicians, can help identify strategies to recruit potential participants.

### Choosing a data collection method

The choice of methodology will dictate the methods of data collection available to researchers.^13^ While the benefits and drawbacks of using different methods are well described,^13,25,49^ applying them to study AMS highlights unique challenges and opportunities. We focus here on interviews and focus groups as the most commonly used methods of data collection in qualitative research.^49^ Interviews are a rich source of information, and are particularly useful in gaining an insider’s perspective or when talking about sensitive topics.^49,50^ In contrast, focus groups rely on interaction between participants and seek to unearth a shared understanding; they are suitable for exploring diversity and consensus in views, beliefs and attitudes on a particular topic.^51^ Focus groups can provide a more realistic environment for expressing views than interviews as in real life we are also influenced by others.^52^ Given multiple actors involved in AMS, it is worth considering how focus groups or interviews may make a contribution to this field and when they can be most suitable.

First, when the focus is on individual experiences^53^ interviews may be a more suitable method. For example, while patients’ views about antibiotic prescribing could potentially be gathered through both interviews and focus groups, patients’ experiences of managing their own symptoms and help-seeking behaviour in relation to specific infections are often best accessed through interviews. Second, both focus groups and interviews may be suitable for exploring sensitive or controversial topics, but need consideration. On the one hand, interviews may be considered as more appropriate in facilitating rapport; on the other hand, if participants feel they can discuss their views without judgement from other participants or the facilitator focus groups may also be suitable.^49^ For example, when focusing on a particular infection, it is worth thinking about what characteristics may impact the group dynamics. In consideration of this, Duane *et al.*^42^ decided to conduct separate focus groups with female and male participants in relation to their views of UTIs, as they wanted to avoid potential embarrassment due to the topic. The importance of group dynamics and extent of group diversity can also be important when doing research with HCPs. For example, a study on views of general practice staff on two AMS strategies involved focus groups, with each having at least two prescribers and other clinical and administrative staff.^18^ This diversity in the composition of the groups allowed participants to discuss and address different views and experiences, but the presence of colleagues with different roles might have influenced what individuals shared and, in some instances, led to a dominance of doctors’ views.^18^

We also acknowledge observation as another method of data collection and a useful way of understanding the meanings, cultural practices and actions of people in their everyday context, thus focusing on what people do rather on what people say they do.^13^ It can be an important way of gathering insight into how people behave and engage with different practices related to AMS/AMR, as discussed in depth elsewhere in this series.^54^ Broadly speaking, observation methods can be divided into participant observation (where the participant is actively participating in the setting and observed activities)^55^ and non-participant observation (where the researcher does not participate in the activities being observed and thus takes more of a distant role).^55^ A recent development in the area of non-participant observation includes an increasing use of video recordings.^19,33,34^ This can be a useful data collection approach in primary care research particularly when studying patient–clinician communication practices related to AMS.^34,35,56^

### Conducting data analysis

First, data analysis will be strongly linked to the methodology chosen. For example, despite commonalities across experiential approaches, the approach to analysis in grounded theory or phenomenological approach will, or should, look slightly different. While in grounded theory study we may aim to develop a meta-theory from the empirical data of why GPs may prescribe broad-spectrum antibiotics,^57^ a phenomenological study would be more suited to questions around nurses’ views and sense making of antibiotic prescriptions.^58^ These differences at times may be subtle and will be closely linked to the scope and aims of each methodology. Understanding what each methodology can offer is important in ensuring that researchers choose the most appropriate one for answering the research question and ultimately providing findings that can have an impact on clinical practice. Second, researchers may want to consider whether inductive analysis (analysing data without using an existing coding framework or theory^59^), deductive analysis (using existing coding frameworks or theories to guide data analysis^59^) or combined inductive and deductive approaches is more suitable. Inductive data analysis is used widely in AMS qualitative research, with deductive analysis less common. For example, Cabral *et al.*^33^ used a comprehensive coding scheme, created based on findings from previously published conversation analysis studies, and deductively coded the majority of their transcripts using this framework. Combining inductive and deductive approaches can also be useful in order to explore views while in addition drawing on a relevant theory.^60,61^ For example, Courtenay *et al.*^60^ focused on understanding influences on antibiotic prescribing by nurses and pharmacist prescribers; they inductively coded interviews but then mapped these codes onto an existing theory, thus allowing a systematic examination of the influences.

Third, both researcher triangulation and method triangulation can be of particular benefit to AMS qualitative research. Researcher triangulation refers to two or more researchers in the same study being involved in the analysis and interpretation of findings.^62^ While the initial focus of researcher triangulation was to ‘avoid’ bias or as a confirmatory strategy, it is now more recognized that it can enhance the quality of the research by bringing out and celebrating different perspectives. In AMS qualitative research it may be particularly beneficial to have both clinical and non-clinical researchers contributing to data analysis. For example, clinicians’ understanding and experience of clinical guidelines and practice can be helpful in making sense of participants’ narratives and making clinically relevant and feasible recommendations based on study findings. On the other hand, non-clinical researchers can bring to analysis their understanding of an issue through a theoretical lens. Altogether, this can result in pluralistic insights and illuminate the problem from diverse angles.^63^ For example, Björnsdóttir and Hansen^64^ described the value of having pharmacists and sociologists working together on their analysis.

Data triangulation can also be valuable, i.e. using multiple data sources to gain a more holistic understanding. For example, it can help to understand how diverse actors perceive a particular issue related to AMR. Biezen *et al.*^29^ conducted interviews and focus groups with GPs and parents about their perceptions regarding antibiotic prescribing for RTIs in young children. They analysed data together and presented one set of themes for both groups, thus highlighting both convergence and divergence in parents’ and GPs’ views. Data triangulation can also offer crucial insights on how the same intervention can be used and perceived by different actors. For example, Tonkin-Crine *et al.*^65^ compared patients’ and HCPs’ views gathered through interviews as part of the trial process evaluation and identified important similarities and differences in how these two groups perceived the benefits of point-of-care C-reactive protein tests and the related antibiotic prescription decisions. It is worth highlighting that qualitative and quantitative data can also be brought together and offer complementary perspectives.^65^ For example, Strandberg *et al.*^66^ combined quantitative data (prescription data, patient questionnaires and GPs’ audit) and qualitative data (patient observation and interviews) in order to identify and understand factors affecting antibiotic prescribing.

### Importance of patient and public involvement

Patient and public involvement (PPI) can be defined as an active partnership between patients, the public and researchers as part of the research process, where research is conducted ‘*with* or *by* patients’ rather than ‘*to* or *about* them’.^67^ PPI in AMS research can be seen not only as a marker of good quality,^68^ but also further highlighting the value of multidisciplinary approach to conducting AMS research.

In the initial stages, PPI input can ensure that the research topic is relevant and of importance to patients or public,^69,70^ that methods are appropriate and recruitment materials understandable to the potential participants.^71,72^ As the study progresses, PPI representatives can advise on identifying and optimizing recruitment strategies.^73^ They might also act as gatekeepers for recruitment providing access to networks and newsletters, charities and other associations, which can be particularly beneficial. In the later stages they can inform analysis; for example, multidisciplinary teams in the analysis may also include PPI members and in the later stages, ensure that findings are understandable and actionable and reach the right audiences.^74,75^

Beyond this, there may be a need for more systemic and structural approach to developing PPI activities; for example, rather than seeking PPI input for discreet AMS research projects, it is worth thinking how we can develop relationships long term and build institutional infrastructure for conducing meaningful PPI input in AMR and AMS field. This may be achieved through local and global initiatives but needs organizational commitment and leadership and long-term resourcing.^76^ Developing a strategy that allows long-term relationships may be particularly beneficial to ensure that research which is relevant to patients and public is prioritized and conducted.

## The importance of ensuring quality in qualitative research

The topic of quality in qualitative research has received substantial attention over the last two decades, yet the debates on what good qualitative research should look like are very much ongoing. As recently summarized by Pope and Mays,^25^ the three main viewpoints seem to be: (i) rejection of the need for quality criteria for qualitative research; (ii) the need to use the same quality criteria as in quantitative research; and (iii) the need to identify and apply separate quality criteria for qualitative research. Furthermore, recent voices suggest that we may in fact need criteria for each qualitative methodology.^77^ For example, different papers highlight what good qualitative research may look like in a study using phenomenological approaches,^78^ thematic analysis,^79^ ethnography,^80^ grounded theory,^81^ conversation analysis^82^ or discursive psychology.^83^

While we do not attempt to resolve these tensions here, nor we are able to, it is important that the primary care AMS research community joins these debates. For researchers, it may mean ensuring that they contribute to these debates as discussions on what it means to apply the quality criteria to qualitative research in AMS and primary care are largely missing. This may mean publishing methodological and theoretical discussions around the quality of qualitative research in medical journals, rather than those specifically dedicated to qualitative research. The multidisciplinary nature of AMS research permeates a variety of aspects key to quality issues that AMS researchers should be aware of. If qualitative and quantitative AMS researchers are to work together, there is a need to establish how diverse expertise is (best) utilized in data analysis. Equally, it is important that researchers are aware of the importance of reflecting on individual assumptions brought to the project and analysis (as part of reflexivity). It is also crucial that these aspects are reported in published papers, within methods sections, and are expected by the AMS community, which requires a paradigm shift. A recognition of issues around reflexivity, which is key to ensuring both high quality and ethical studies, is rare. Notable examples of these include reflections on the impact of professional roles (GP versus social sciences researcher) on qualitative interviewing,^84^ or on interviewing fellow healthcare professionals;^85^ these can be powerful and well received by primary care community.^84^

For journal editors and peer reviewers, it may mean that they are aware of the tensions and debates on the quality and ensure that these are reflected in how they view qualitative submissions and, most importantly, ensure that the debates around quality find home in the medical journals as well. These issues are of course not unique to AMS and primary care research but highlight some directions towards ensuring that qualitative research is seen at par with their quantitative counterpart.

## Contribution of qualitative research to primary care

Throughout previous sections, we described practical considerations when conducting qualitative research, while highlighting how it can contribute to AMS, drawing on examples from relevant studies. Here, we make a further case, summarizing the three key areas where qualitative research can make a difference to tackling AMR and promoting AMS. In meeting these overarching aims, qualitative research fits well with using behavioural^86^ and social science^87^ approaches to addressing AMR/AMS.^87^

First, qualitative research allows us to describe and understand the problem of AMR from the perspectives of the relevant stakeholders and the meanings they attach to it. This may mean focusing on understanding how patients, HCPs or policymakers perceive AMR as an issue in itself, thus allowing an understanding of the extent to which this is seen as a priority or its relevance (or not) to their lives. It may also mean unpacking specific issues related to AMR, such as understanding patients’ help-seeking behaviour, needs and pressures experienced by patients and HCPs in a consultation, or what makes it difficult to manage certain infections. Second, these insights can crucially help us identify what needs to change, and how relevant actors need to be supported.^38^ This in turn can inform development of AMS programmes through ensuring that they are tackling the ‘right’ problem and are targeting the right people. Third, once interventions are developed and then trialled, qualitative research can contribute to evaluation and understanding of why certain AMS interventions are found to be effective or not, or what specific elements of the intervention work. Qualitative research can illuminate this through exploring how the intervention interacts within its context (primary care), what the key uncertainties related to the intervention are, and how they are received by relevant stakeholders,^88^ and thus how the intervention can be further refined. It can also help us understand how the interventions are received and implemented in the real world.^14^

## Future directions and recommendations for qualitative research in primary care and AMS

Qualitative research in AMS/AMR faces numerous challenges and opportunities in making an impact in the AMS/AMR field. Table 3 provides key recommendations for relevant stakeholders to ensure maximum impact from qualitative research. First, while AMS was a global priority before the COVID-19 pandemic, there has been increased recognition of the long-term global threat of AMR as a result.^89^ This has been linked to the use of broad-spectrum antibiotics in the management of COVID-19, as well as AMS not being seen a priority in the context of a (different) healthcare emergency.^90^ WHO has also highlighted how the pandemic can impact AMS activities and drive AMR through multiple routes, including inappropriate prescribing, hospital admissions increasing the risk of healthcare-associated infections, or disruption to healthcare services.^91^

**Table 3.** Recommendations in relation to key issues for qualitative research in AMS

Issue	Recommendations
Need for AMS qualitative researchers and policymakers to work together to ensure translation of knowledge into policy and practice	Ongoing dialogue between qualitative researchers and policymakers in order to identify critical research questions which qualitative research can answer and how the findings can be best used to inform policy
Involvement of more diverse actors in AMS research	Sampling needs to reflect an increasing diversity in the primary care workforce and other, previously overlooked stakeholders (e.g. policymakers)
Need for developing the field of qualitative methods to answer relevant AMS questions	AMS qualitative researchers need to gain a greater understanding of the pros and cons of diverse methods of data collection, including remote methods and rapid methodologies, previously underutilized in AMS research
Need for multidisciplinary collaboration	Ensure that qualitative research teams have members representing a variety of disciplines
	Ensure that qualitative research is integrated within wider programmes and published together with quantitative work, drawing on integration techniques from mixed methods literature to facilitate a greater understanding of an issue

In primary care specifically, the COVID-19 pandemic added other steps to managing infections and consequently AMR; for example the nature of the COVID-19 symptoms and the use of remote care brought uncertainties in how to manage patients with RTIs and made decision processes on whether to prescribe antibiotics or not more challenging.^92,93^ While some of these may have become a ‘new normal’, the long-term impact of these changes will still need to be explored. Qualitative research can contribute to understanding these changes through, for example, studying patients’ and clinicians’ views of managing infections in the post-pandemic world, views on AMR, the barriers and facilitators to use of point-of-care tests and the impact of disrupted services on delivery of care or help seeking. It is important that the value of qualitative methods in exploring these topics is recognized by the AMS community as well as policymakers, to ensure translation of knowledge into policy and practice.

Second, as highlighted before, with increasing diversity in primary care workforce, there is a need for more studies exploring views of diverse actors related to AMS. Cultural differences between healthcare professionals may shape knowledge, beliefs and norms and may result in variation in care.^94^ Understanding these is crucial in order to develop AMS interventions targeted at these groups.^95^ Equally, understanding the views of policymakers could help develop interventions to improve the healthcare system as well, as these may have been overlooked.^95^

Third, qualitative methodologies have also been facing new challenges and opportunities. While even pre-COVID-19 pandemic technology was used to collect data,^96^ qualitative researchers have rapidly implemented remote data collection methods during the pandemic in order to improve the safety of researchers and participants.^96^ Remote data collection may be less needed in the future, but greater understanding of the pros and cons of these methods of data collection gained during this pandemic (e.g.^97–100^) may encourage researchers to use them beyond this healthcare emergency. It can potentially also increase the use of methods underused in (AMS) qualitative research, including written or video diaries, photovoice or analysis of print or broadcast media (and many others).^101^ Thus, remote methods of data collection may in the future become AMS researchers’ choice rather than the necessity.

Also, current and previous pandemics have highlighted the need for rapid data collection, analysis and sharing of findings in order to promptly inform changes to policy and practice.^102^ This has been reflected in the increasing use of rapid qualitative techniques, such as analysing data from recordings (rather than transcripts) or using a group approach to data analysis.^103^ Rapid data collection and analysis are not new, but to date have been underutilized in AMS. Qualitative research conducted rapidly can make a valuable contribution to understanding issues that are more time sensitive or that would benefit from rapid dissemination. For example, exploring patients’ views of diagnostic tests when they are first introduced (rather than retrospectively) or clinicians’ experiences of a change in antibiotic prescribing practices (e.g. introduction of a new guideline) may benefit from rapid data collection, findings from which can be fed back to policymakers. Equally, some aspects of AMR and AMS, such as long-term impact of COVID-19 on prescribing behaviour, may benefit from longer fieldwork and longitudinal study designs.^104^ AMS researchers will ultimately need to make decisions on what methodologies, methods and timelines are appropriate for each project in order to make a valuable contribution. While it may not be feasible for each member of a research team to be versatile in all qualitative methodologies, it is important that research teams have sufficient expertise to make sound methodological decisions.

Finally, for both qualitative research and AMS to truly make its contribution, there is a need for multidisciplinary collaboration. First, this needs to be reflected in the composition of the research teams; we urgently need researchers from a variety of disciplines working on studies using qualitative methodologies. Second, we also need qualitative research to be integrated within wider programmes of work, using more integrated study designs, where different methods are seen as enriching our understanding of the problem rather than not being compatible with each other. A recent review highlighted the need for evaluations of AMS programmes to be complemented by social sciences and qualitative methods.^105^ Integration of quantitative and qualitative data is still rare, a challenge not specific to AMS^106^ and some have called for more guidance on integrating different approaches that in turn could help not only researchers but also funding boards, reviewers and readers.^106^ For this to happen, we also need mutual learning and integrated working, with qualitative researchers also understanding the value and contributions of other methodologies and disciplines. Others also highlighted the challenges of cross-interdisciplinary research with researchers representing different disciplines needing to be aware of the politics of knowledge production and the need to engage in a dialogue about legitimacy of different methods.^107^ Ding, Pulford and Bates^108^ highlighted that these are not easy tasks as cross-disciplinary collaboration requires commitment from individuals, teams, institutions and funders. While understanding specific challenges to AMS in primary care is crucial, there is a need for an engagement with a One Health approach and unity of variety of stakeholders from multiple sectors working towards the same goal.^89^ There is still work needed to bridge the gap between different disciplines including biomedical, social, environmental and animal sciences.^105,109^

## Conclusions

Qualitative research can make a valuable contribution to AMS/AMR research, despite its value at times being questioned and misunderstood. Qualitative research can help us to understand the problem from the perspective of relevant stakeholders, use this understanding to develop AMS interventions, and then study how these interventions are implemented and received in formal evaluations and beyond. Through highlighting unique considerations when planning and conducting qualitative research on AMS in primary care, we hope to ensure that researchers use these methods, and that qualitative research can achieve its full potential as standalone or in mixed-methods research.
